# Development of high-resolution daily gridded temperature datasets for the central north region of Egypt

**DOI:** 10.1038/s41597-019-0144-0

**Published:** 2019-07-31

**Authors:** Mohamed Salem Nashwan, Shamsuddin Shahid, Eun-Sung Chung

**Affiliations:** 1 Construction and Building Engineering Department, College of Engineering and Technology, Arab Academy for Science, Technology and Maritime Transport (AASTMT), 2033 - Elhorria, Heliopolis, Cairo Egypt; 2 School of Civil Engineering, Faculty of Engineering, Universiti Teknologi Malaysia (UTM), 81310 Skudai, Johor Malaysia; 3 Department of Civil Engineering, Seoul National University of Science and Technology, Nowon-gu, 01811 Seoul South Korea

**Keywords:** Projection and prediction, Hydrology

## Abstract

This study developed 0.05° × 0.05° land-only datasets of daily maximum and minimum temperatures in the densely populated Central North region of Egypt (CNE) for the period 1981–2017. Existing coarse-resolution datasets were evaluated to find the best dataset for the study area to use as a base of the new datasets. The Climate Prediction Centre (CPC) global temperature dataset was found to be the best. The CPC data were interpolated to a spatial resolution of 0.05° latitude/longitude using linear interpolation technique considering the flat topography of the study area. The robust kernel density distribution mapping method was used to correct the bias using observations, and WorldClim v.2 temperature climatology was used to adjust the spatial variability in temperature. The validation of CNE datasets using probability density function skill score and hot and cold extremes tail skill scores showed remarkable improvement in replicating the spatial and temporal variability in observed temperature. Because CNE datasets are the best available high-resolution estimate of daily temperatures, they will be beneficial for climatic and hydrological studies.

## Abstract (summary)

**Table:** 

Design Type(s)	time series design • modeling and simulation objective
Measurement Type(s)	temperature
Technology Type(s)	computational modeling technique
Factor Type(s)	geographic location
Sample Characteristic(s)	Egypt • climate system

Machine-accessible metadata file describing the reported data (ISA-Tab format)

## Background & Summary

**Fig. 1:**
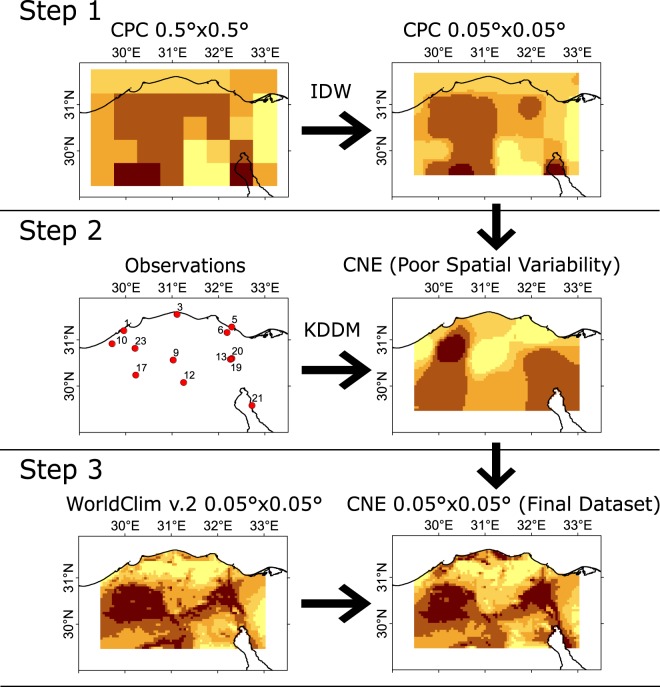
The three-step methodology adopted to generate the high-resolution CNE datasets of maximum and minimum temperatures. The first step was the interpolation of raw CPC data to a higher resolution. Then, the observation data were used to bias correct the high-resolution CPC using KDDM. Finally, the WorldClim v.2 temperature climatology was used to adjust the spatial variability in the CNE data.

Regularly gridded meteorological observation data are important for climate analyses^1^. Although many high-resolution gridded meteorological datasets are already available for other regions^1–7^, Egypt has none. This study newly developed gauge-based gridded datasets provide bias-corrected, high-spatial-resolution (0.05° × 0.05°) and relatively long-term record (37 years) land-only daily maximum and minimum temperatures. They are available for the Central North region of Egypt (CNE) (latitude: 29.50°–31.55°; longitude: 29.50°–33.00°), where more than 70% of Egyptians live^8^, more than 60% of the Egyptian agricultural land is cultivated^9^, and most of the industrial activities are located^10^. The availability of these data is important for several reasons. First, as they are evenly gridded data, they provide the best available high-resolution estimates of daily surface maximum and minimum temperatures in a data-sparse region where observation stations are limited. Second, these data will be beneficial in validating regional climate models for the better prediction of climate change. Third, any climate change impact model usually requires evenly spaced temporally complete meteorological data, which can be served using these data. Therefore, it will enable both hydrologists and meteorologists to enhance their assessments of daily scale hydrological hazards, such as heat and cold waves. Fourth, they will give bias-free monitoring of climate change and variability at a fine resolution using difference indices and help in the comparison of the regional rate of change to the global rate. Prior to the data development, the existing gauge-based coarse-resolution datasets were evaluated using different statistical indices to find which of them can better estimate the observations of stations in the study area. The dataset found to be statistically better in estimating the observed temperature was selected as the base of the newly developed data. Figure 1 shows an overview of the three-step methodology used to develop the CNE datasets. The selected dataset, Climate Prediction Center (CPC) global temperature, was interpolated to generate high-resolution data. Then, the new robust kernel density distribution mapping (KDDM)^11^ method was used to correct the bias of high-resolution data using daily observations. Finally, WorldClim v.2 temperature climatology was used to adjust the spatial variability in the maximum and minimum temperatures of the newly developed dataset^12^. The non-stationarity, trend and extreme values were taken into consideration when developing the CNE datasets.

The performance of the newly developed CNE datasets was validated in terms of their ability to replicate spatial and temporal variability in temperature and its distribution and extremes. The CNE datasets showed a remarkable improvement in the replication of the spatial and temporal variability in observed daily maximum and minimum temperatures (Tmx and Tmn, respectively); diurnal temperature range (DTR); and monthly means of Tmx, Tmn and the mean temperature (Tm is estimated as (Tmx + Tmn)/2). Furthermore, the probability density function (PDF), skill score (PDF_SS_) and hot and cold extreme tail skill scores (tail_SS_) showed that the CNE is more capable compared to other available datasets in reproducing the observed data distribution and extremes. The CNE datasets are freely available online in NetCDF format^13^. It can be argued that the CNE datasets are the best available high-resolution, gauged-based estimates of daily near-surface temperatures in such a data-scarce region.

## Methods

### Data

In this study, six gridded datasets were used. They are the Climate Prediction Center (CPC) global temperature, the Princeton University Global Meteorological Forcing (PGF) v.1, WorldClim v.2, University of East Anglia Climatic Research Unit Time Series (CRU TS) v4.01, University of Delaware (UDel) Air temperature v5.01 and the Climatologies at High resolution for the Earth’s Land Surface Areas (CHELSA) v1.2. The CPC and PGF datasets were used in the predevelopment evaluation process. They are the only available gauge-based, gridded daily Tmx and Tmn datasets for the study area. WorldClim v.2 was used to adjust the spatial variability in the new data. The CRU, UDel and CHELSA datasets were used, along with the station data, to validate the new data. A summary of the six gridded datasets used in the present study is given in Table 1, and a brief description of each one is given below.

**Table 1:** Summary of the gridded datasets used in developing or validating the CNE datasets.

Dataset	Spatiotemporal Resolution	Temporal Span	Source
CPC	0.5°, daily	1979-present	^37^
PGF v.1	0.25°, daily	1948–2010	^38^
WorldClim v.2	2.5 arc minute, monthly climatology	1970–2000	^39^
CRU TS v4.01	0.5°, monthly	1901–2016	^40^
UDel v5.01	0.5°, monthly	1901–2017	^41^
CHELSA v1.2	30 arc second, monthly time series and climatology	1979–2013	^42^

The CPC dataset has been developed by the American National Oceanic and Atmospheric Administration (NOAA) using the optimal interpolation of quality-controlled gauge records of the Global Telecommunication System (GTS) network^14^. The PGF dataset has been developed by assimilating the National Center for Atmospheric Research reanalysis datasets with several global observation databases^15^. The high-resolution, 2.5 arc minute, WorldClim version 2 has maximum and minimum temperature climatology gridded data. It has been developed by thin-plate spline interpolation of weather station data. The interpolation covariates were elevation, distance to the coast, and MODIS satellite data (day and night temperate and cloud cover)^12^. WorldClim has a global cross-validation correlation of more than 0.99^12^. Therefore, it was used to adjust the spatial variability in the new data. The CRU gridded data have been developed using the angular distance weighting interpolation of monthly observed data obtained from the World Meteorological Organization (WMO), NOAA and other national-level observed datasets^16^. The UDel dataset has been developed using the climatologically aided interpolation^17^ of the Global Historical Climatology Network dataset, the US National Climate Data Center Global Summary of the Day (GSOD) dataset, and selected station data from the Legates and Willmott^18^ archive. The CHELSA maximum and minimum temperature climatology and time series are statistical downscaled model outputs of the ERA-Interim reanalysis data at a 30 arc second spatial resolution for 1979–2013^19^.

**Fig. 2:**
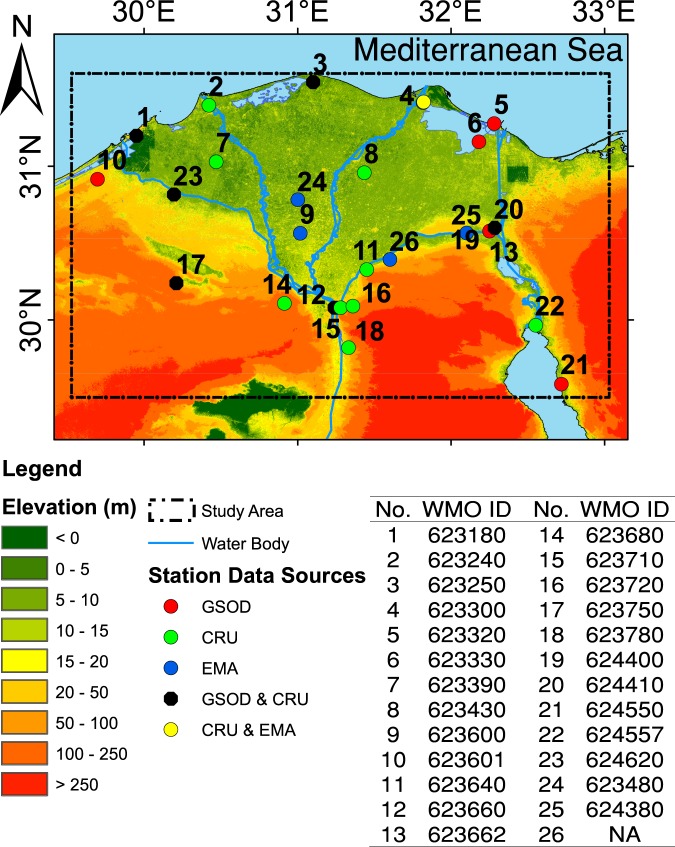
A map of the central north region of Egypt. The map shows the boundary of the newly developed data (latitude 29.50°–31.55° and longitude 29.50°–33.00°), the locations of the 26 observation stations used in the study, and the ground elevation.

Daily observations of Tmx and Tmn at 12 stations were obtained from the GSOD dataset for the study period 1981–2017 (Fig. 2). In addition, observation data from four stations were acquired from the Egyptian Meteorological Authority (EMA). The observations at station nos. 4, 24, 25, and 26 were used for the validation of CNE data, while the remaining observations from 13 stations were used for the development of the dataset. Several quality checks were carried out to ensure the homogeneity of the observed data. Furthermore, quality-controlled monthly averages of observed Tm at 16 stations were obtained from the CRU TS v4.01^20^ database for the validation of CNE datasets (Fig. 2).

### Data development

**Fig. 3:**
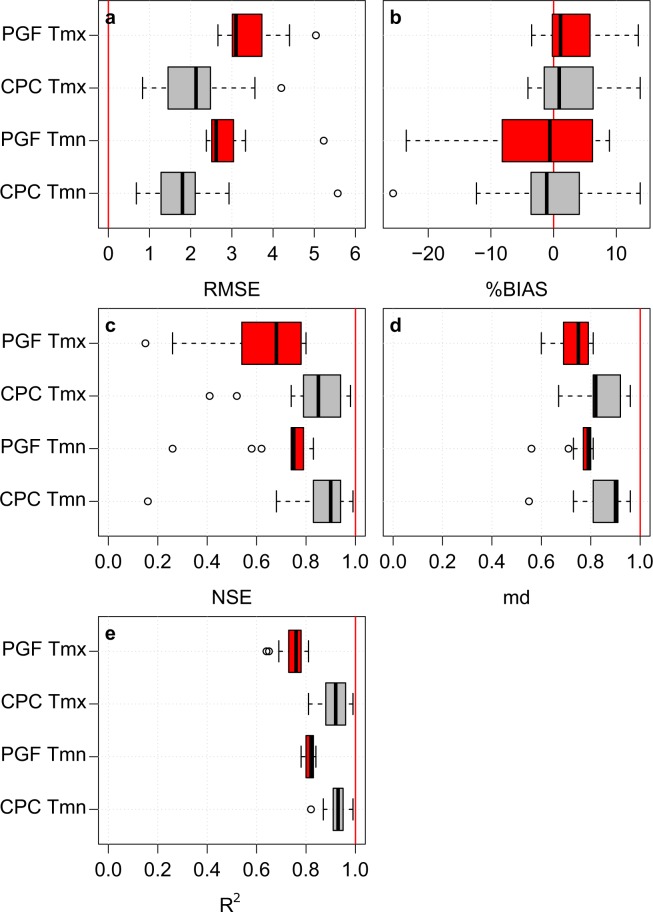
The predevelopment evaluation results of the gauge based daily Tmx and Tmn datasets of CPC and PGF. The (**a**) RMSE, (**b**) %BIAS, (**c**) NSE, (**d**) md and (**e**) R^2^ were calculated at each station separately and are presented as box plots. The red vertical line in each plot panel represents the optimum value of the corresponding index. It can be seen that the CPC dataset is better than the PGF dataset in estimating the daily Tmax and Tmin.

Prior to the CNE dataset development, the performances of the CPC and PGF datasets were evaluated to determine which of them is better in estimating the observed daily Tmx and Tmn. For this purpose, the CPC and PGF data were interpolated at each of the 13 station’ locations using inverse distance weighting (IDW). The daily assessment was based on five statistical indices, namely, root mean square error (RMSE), the percentage of bias (%BIAS), Nash-Sutcliffe efficiency^21^ (NSE), modified index of agreement^22^ (md), and coefficient of determination (R^2^). The RMSE measures the differences between the observed and the gridded time series. The %BIAS measures the range of the average tendency of the gridded time series against the observed time series. The optimal value of RMSE and %BIAS is zero. The NSE determines the relative magnitude of the residual variance in the gridded data compared to the variance in the station data. The md estimates the additive and proportional differences in the means and variances of the observed and gridded data. Finally, R^2^ assesses the degree of collinearity between gridded and observed data. The last three indices have an optimal value of one. Supplementary Table 1 presents the formula of each index and its value range. The above indices have been widely used for the evaluation and validation of gridded data^3,5,23,24^. They were calculated at each station separately. The obtained results are presented as a box plot in Fig. 3. The figure indicates a better performance of CPC compared to PGF for most of the indices at all 13 stations. Although it has a large bias, the CPC dataset was chosen as the base for the development of the new high-resolution data.

CPC showed random errors and bias when compared with the observations, which may result from insufficient *in situ* data coverage and imperfection in data assimilation and interpolation^16^. Therefore, it was required to correct the bias of the CPC data. There are several methodologies available for bias correction in meteorological time series^25,26^. A new robust approach named KDDM, which was developed by McGinnis, *et al*.^11^, was used in the present study. In core, it is not different from the most widely used probability mapping bias correction method^27^, except that it uses a nonparametric estimate of the underlying PDFs instead of using a fitted parametric distribution. KDDM has been used in several studies^28,29^ and found to be the best approach of daily bias correction when compared to others^11^.

The methodology adopted in this study was structured using the following steps (demonstrated in Fig. 1): (1) the 0.5° × 0.5° CPC Tmx and Tmn datasets were regridded to a 0.05° × 0.05° spatial resolution using the IDW method; (2) the KDDM bias correction was applied to correct the bias in daily temperature data against the observed data; and (3) the spatial variability in temperature from the regridded data were corrected using the WorldClim v.2 temperature climatology^12^, which is available at a 2.5 arc minute spatial resolution. The WorldClim monthly means were corrected using station data to consider the global temperature rise in recent years. Further details of the bias correction are provided below.

For each 0.05° grid point, a search for the nearby available stations within a threshold distance was conducted. The observation data of the stations found within the threshold distance were interpolated to the grid point using IDW. The interpolated time series and the corresponding grid time series were normalized separately using Z-score. The normalization was performed for each one-month climatological window separately. The Z-score was selected for normalization, as it considers both the mean value and the variability in the raw dataset by preserving the range (maximum and minimum values) and introduces the dispersion in data. This approach also separates the nonstationary climate change signal from the bias in the shape of the distribution. The KDDM bias correction was conducted over these normalized data. The KDDM estimates the kernel density of the distribution of both the normalized CPC (nCPC) and the normalized interpolated time series data (nObs) based on the monthly climatological windows. The kernel density was calculated based on the default Gaussian kernels^30^, and the bandwidth was selected using Silverman’s rule of thumb^31^. The nonparametric PDFs of both nCPC and nObs were numerically integrated to calculate the cumulative density functions (CDFs) by applying the trapezoidal rule and fitting a spline to the corresponding quantiles. Later, a transfer function was applied by combining the forward CDF of nCPC and the inverse CDF of nObs using Eqs (1) and (2), respectively.

1 $$P(x)=\int \frac{1}{n}\sum _{i=1}^{n}{K}_{h}(x-{x}_{i})dx$$

2 $${x}_{bc}={P}_{nObs}^{-1}\left[{P}_{nCPC}({x}_{nobs})\right]$$

where *P*(*x*) is the CDF of time series *x* n is the number of data points, *K*_*h*_ is the kernel function scaled to an h bandwidth, and *x*_*bc*_ is the resulting bias-corrected time series.

Finally, the bias-corrected 0.05° grid point time series were denormalized to generate the bias-corrected data at each grid point.

## Data Records

The data records of the daily high-resolution (0.05°), land-only, near-surface maximum and minimum temperatures, in °C, for the CNE for the period January 1981 to December 2017 are freely available online within Figshare^13^ in NetCDF format. The data records spatially cover the land area bounded by latitudes of 29.50° and 31.55° and longitudes of 29.50° and 33.00°. The records will be updated frequently in the upcoming years when more observation data will be available. The spatial coverage of the CNE data may be extended in the future when more observation data are available.

## Technical Validation

The newly developed CNE datasets were validated at different time scales in four steps. First, the performance of CNE with respect to the CPC and CRU datasets was assessed according to their abilities to replicate the daily observed temperature at 13 stations that were used during data development. Second, the CNE datasets were validated using independent station data. In the third step, the CNE dataset was validated against the monthly mean temperature from the CRU TS v4.01 station data. Finally, the spatial variability in the CNE datasets was validated against the high-resolution CHELSA dataset. Overall, the CNE showed remarkable performance.

### Validation of the daily and monthly maximum and minimum temperatures

**Fig. 4:**
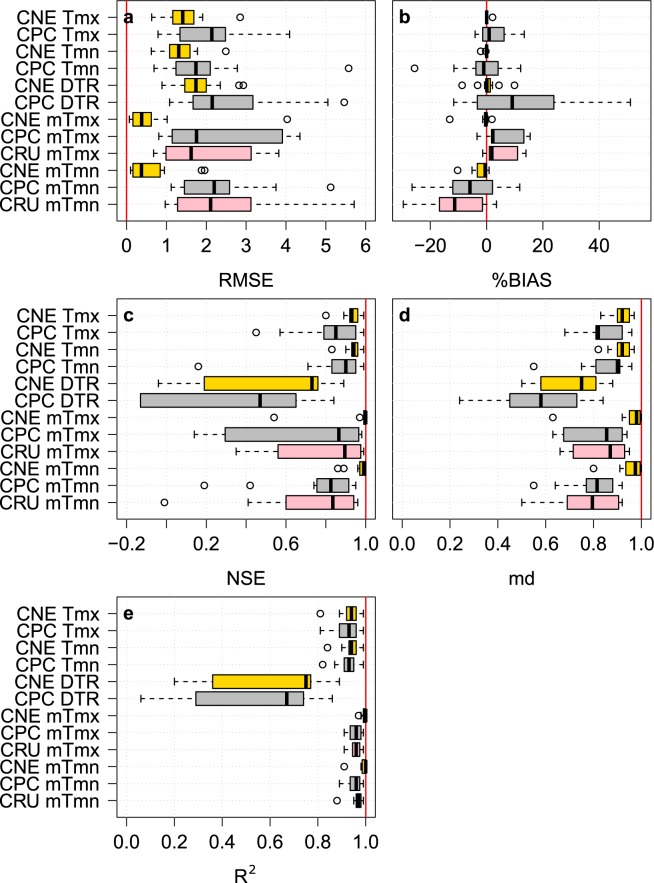
The performances of the CNE datasets compared to the CPC and CRU datasets in replicating daily and monthly maximum and minimum temperatures. Box plots of the five statistical indices (**a**–**e**) used to show the performances of the CNE, CPC, and CRU compared to the observed Tmx, Tmn, DTR, mTmx, and mTmn at 13 stations. The red vertical line in each plot panel represents the optimal value of the corresponding index.

The performances of daily Tmx, Tmn, and DTR of CNE were compared to those of CPC datasets at 13 stations that were used for the development of the CNE datasets. In addition, the monthly average maximum and minimum temperatures (mTmx and mTmn, respectively) at the same stations were calculated and used to verify the performance of the CNE data compared to that of the CPC and CRU data. At each station, the CNE, CPC, and CRU were interpolated to assess their performance with respect to the observations. Five statistical indices were used (RMSE, %BIAS, NSE, md, and R^2^) to evaluate the performance. The performance was assessed at each station separately, and the results of each index are presented as box plots in Fig. 4.

The validation results show that the CNE had a median RMSE value of 1.41 (1.31) for Tmx (Tmn), which was much better than the CPC values (Fig. 4a). The median %BIAS of CNE was zero for both Tmx and Tmn. The median NSEs for CNE were 0.93 and 0.94, and the mds were 0.92 and 0.92 for daily Tmx and Tmn, respectively, which were much higher compared to the CPC values. The medians of R^2^ of the CNE were found to be very close to those of the CPC, however, the minimum whiskers of the box plots of R^2^ for the CNE were better.

Because the DTR is an important index that can provide a spatial fingerprint of climate change^32,33^, it should be accurately estimated^34^. The DTR estimated by the CNE was more accurate than that by the CPC when compared to observations using the performance indices. The RMSE of CNE had a median of 1.74, with a relatively narrow range of RMSE compared to that of the CPC. The DTRs estimated by CPC were heavily overestimated, with a median %BIAS of 9.1, while the median %BIAS for CNE was 0.1. The CNE scored median NSE, md and R^2^ values of 0.73, 0.75, and 0.75, respectively, which were better than those of CPC (0.47, 0.58, and 0.67, respectively).

At the monthly scale, the CNE outperformed both the CPC and CRU in replicating mTmx and mTmn. The median RMSE of CNE was 0.38 for mTmx and mTmn, while they were approximately 1.7 and 2.1 for mTmx and mTmn for both the CRU and CPC, respectively. Similar to daily Tmx and Tmn, the %BIAS of mTmx and mTmn of CNE were nearly zero, but CPC and CRU showed a wide range of positive and negative biases, especially for mTmn (Fig. 4b). The NSE was almost optimal for CNE (near 1), while the medians were 0.87 and 0.83 for CPC and 0.9 and 0.84 for CRU. As shown in Fig. 4d, CPC and CRU showed a large variance in md, ranging between 0.5 and 0.95, while the CNE showed a median value of md and 0.98 for both mTmx and mTmn. CNE had a higher correlation of mTmx and mTmn with the observation data than CPC and CRU.

Next, the accuracy in the distribution of CNE data was assessed using the PDF skill score^35^. The PDF skill score (PDF_SS_) is a robust score that measures the overlap between the modeled and the observed PDFs by computing the cumulative minimum value of their distributions^35^, as in Eq. (3). A perfect overlap between the PDFs is reflected by a score of one. Finally, the tail skill score (Tail_SS_) was used to measure the accuracy of the CNE data to replicate the upper and lower 5% of the observed maximum and minimum temperature PDFs, respectively. Tail_SS_ is a good indicator of matching the extreme values between the two datasets. It begins by calculating the sum of the absolute difference between the upper and lower 5% of the modeled and the observed PDFs. Then, it assigns an increasing weight to the sum of the difference as the temperature values go to the far extreme, as formulated in Eq. (4). Therefore, the 99^th^ percentile (1^st^ percentile) values were weighted more than the 95^th^ percentile (5^th^ percentile). A Tail_SS_ value of one indicates a perfect match between the extreme tails. We calculated the Tail_SS_ for the upper tail of Tmx and the lower tail of Tmn, which represent the extreme hot and cold temperatures, respectively.

3 $$PD{F}_{SS}=\sum _{1}^{n}{\rm{\min }}({Z}_{m},Zo)$$

4 $$Tai{l}_{SS}=\frac{1}{1+{\sum }_{i=1}^{n}{W}_{i}\left|{Z}_{o}^{i}-{Z}_{m}^{i}\right|}$$

where *Z*_*m*_ and *Z*_*o*_ are the ratio of the values for a given *n* number of bins from the modeled and observed PDFs, respectively; *W*_*i*_ is the weight assigned for bin numbered *i*, where the bins are only in the upper or lower tails of the PDFs; and ${Z}_{m}^{i}$ and ${Z}_{o}^{i}$ are the frequency of a temperature value in a given bin for the modeled and observed data, respectively.

**Fig. 5:**
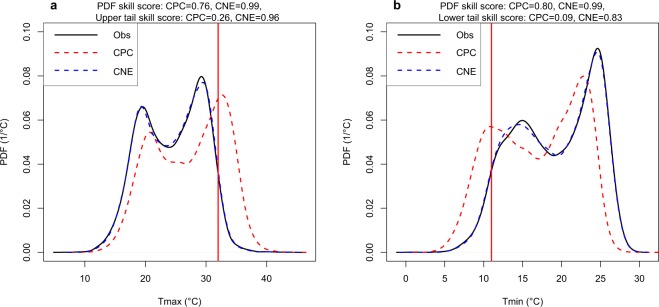
The probability distribution function (PDF) of the observed Tmx and Tmn at station 623330 against the PDFs of CPC and CNE. The PDFs of observation data of (**a**) Tmx and (**b**) Tmn are compared to the PDFs of CPC and CNE data. The red vertical lines represent the 95^th^ and 5^th^ quantiles of observed Tmx and Tmn, respectively. The improvement in the CNE in replicating nearly the same PDFs of the observations is clearly shown compared to the relatively poor performance of CPC, especially for extreme hot and cold temperatures.

An example of the PDF_SS_ and tail_SS_ results obtained is presented in Fig. 5. The figure shows the performances of the CNE and CPC daily Tmx and Tmn for station 623330 during 1981–2017 to replicate the distribution and the extreme values. The PDF of the CNE was found to match better with the observed one compared to CPC. This was also evidenced from the PDF_SS_ values of CNE, which were 0.99 for both Tmx and Tmn compared to 0.76 and 0.80 for Tmx and Tmn, respectively, for CPC. In the case of the hot and cold extremes, which are presented as the ≥95 percentile of Tmx and ≤5 percentile of Tmn, the CNE showed a remarkable performance. The upper and the lower tail_SS_ for CNE were improved by 70% and 74%, respectively, compared to the CPC. Although it seems from Fig. 5 that CPC had a consistent bias in the distribution, this was not the same at other stations. Similar results were obtained at other stations. The comparison of the performances of CNE and CPC in terms of PDF_SS_ and tail_SS_ are presented in Table 2.

**Table 2:** The PDF_SS_ and tail_SS_ values of CNE and the percentage of improvement obtained in CNE compared to the CPC in terms of those indices for both Tmx and Tmn.

Station WMO ID	The CNE PDF_SS_		Percentage of improvement in the PDF_SS_		The CNE tail_SS_		Percentage of improvement in the tail_SS_	
	Tmx	Tmn	Tmx	Tmn	Upper tail of Tmx	Lower tail of Tmn	Upper tail of Tmx	Lower tail of Tmn
623180	0.99	0.99	16%	7%	0.95	0.92	55%	23%
623250	1.00	1.00	8%	7%	0.96	0.95	29%	65%
623320	0.99	0.99	14%	14%	0.94	0.86	51%	73%
623330	0.99	0.99	23%	19%	0.96	0.83	70%	74%
623600	0.98	0.99	28%	13%	0.90	0.83	67%	58%
623601	0.99	0.99	15%	6%	0.93	0.86	34%	32%
623660	1.00	1.00	2%	6%	0.98	0.90	4%	59%
623662	0.96	0.96	1%	2%	0.92	0.55	2%	24%
623750	1.00	0.99	3%	4%	0.99	0.95	12%	61%
624400	0.99	0.99	4%	6%	0.99	0.89	26%	61%
624410	0.99	0.99	7%	4%	0.96	0.71	25%	41%
624550	1.00	1.00	3%	2%	0.99	0.87	22%	20%
624620	0.99	0.98	10%	31%	0.94	0.95	12%	88%

### Validation using independent station data

**Fig. 6:**
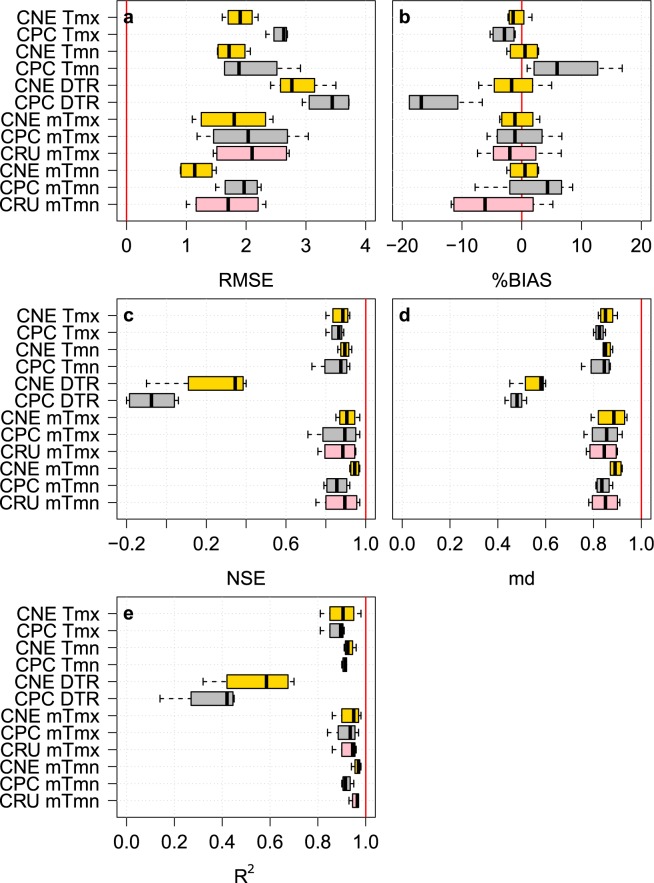
The performance of the CNE datasets compared to CPC and CRU in replicating daily and monthly maximum and minimum temperate at the independent stations that were not used for the development of CNE datasets. Box plots of the five statistical indices (**a**–**e**) used to validate the performances of the CNE, CPC, and CRU datasets compared to the observed Tmx, Tmn, DTR, mTmx, and mTmn at the 4 independent stations. The red vertical line in each plot panel represents the optimal value of the corresponding index.

The independent station data, that were not used for the development of CNE, were used to validate the CNE datasets at the daily and monthly time scales. The stations are nos. 4, 24, 25 and 26 (*refer to* Fig. 2). Overall, the performance of the CNE was found better than that of CPC and CRU in estimating Tmx, Tmn, DTR, mTmx and mTmn. As shown in Fig. 6, the median RMSE value of 1.90 (1.72) for Tmx (Tmn) for CNE was better than that for CPC. CNE had a double-edged %BIAS, with a median of −1.45% and 0.55% for Tmx and Tmn, respectively. CPC underestimated Tmx and overestimated Tmn. The median NSE of CNE was 0.89 for both Tmx and Tmn. Although CPC showed a similar median NSE (approximately 0.85), the NSEs of CNE were closer to the optimal value than that of the CPC. As shown in Fig. 6e, CNE was more correlated with station data than CPC. In the case of DTR, the CNE datasets showed a significant improvement in estimating DTR against CPC, with a median %BIAS of −1.70% and an R^2^ of 0.59.

For the mTmx and mTmn, the performance of CNE datasets was found to exceed those of CPC and CRU (Fig. 6). The medians of RMSE of CNE were 1.80 and 1.14 for mTmx and mTmn, respectively, which were better than those of CPC (2.04 and 2.00, respectively) and CRU (2.10 and 1.70, respectively). The CNE datasets showed the lowest %BIAS and the highest md. The CNE had a higher correlation with the station data than the CPC and CRU for both mTmx and mTmn.

The distribution of CNE daily data was also validated against the station data using the PDF_SS_ and upper and lower tail_SS_. The scores and percentage of improvement in terms of each score compared to the CPC dataset are presented in Table 3. The CNE had a high PDF_SS_ for both Tmx and Tmn, with an improvement of 10%. The upper tail_SS_ of Tmx and lower tail_SS_ of Tmn of CNE were improved by up to 31% and 60%, respectively, compared to those of the CPC.

**Table 3:** The PDF_SS_ and tail_SS_ values of CNE and the percentage of improvement obtained by CNE compared to CPC in terms of the indices for both Tmx and Tmn at the independent stations that were not used for the development of CNE datasets.

Station WMO ID	The CNE PDF_SS_		Percentage of improvement in the PDF_SS_		The CNE tail_SS_		Percentage of improvement in the tail_SS_	
	Tmx	Tmn	Tmx	Tmn	Upper tail of Tmx	Lower tail of Tmn	Upper tail of Tmx	Lower tail of Tmn
623300	0.93	0.96	1%	2%	0.85	0.71	31%	35%
623480	0.95	0.98	10%	4%	0.89	0.80	26%	50%
624380	0.95	0.91	1%	10%	0.83	0.81	17%	60%
NA	0.94	0.95	4%	6%	0.88	0.75	26%	49%

### Validation of the monthly mean temperature using CRU TS v4.01 station data

**Fig. 7:**
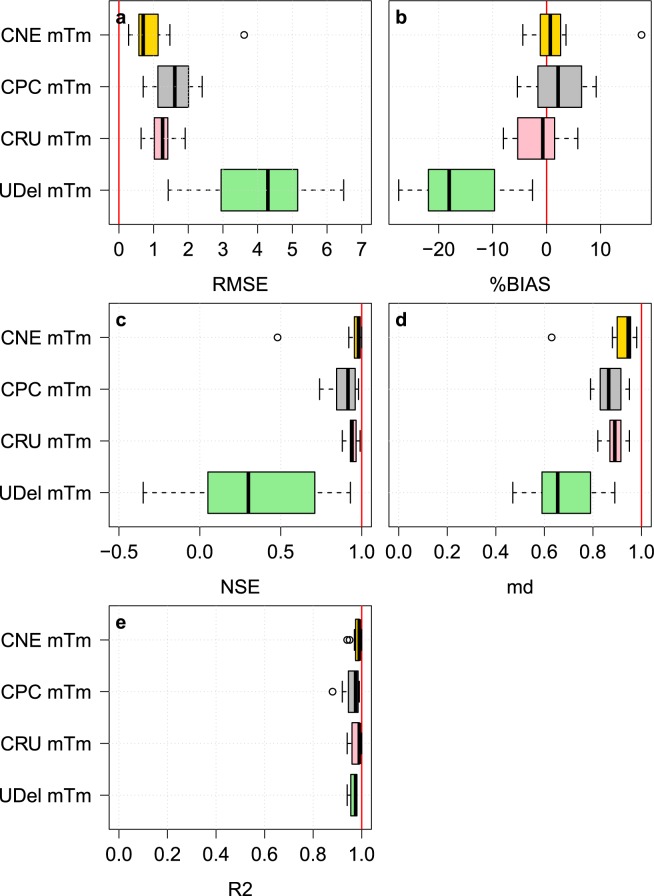
The performances of the CNE datasets compared to the CPC, CRU and UDel datasets in replicating monthly mean temperatures. Box plots of the five statistical indices (**a**–**e**) used to validate the performances of the CNE, CPC, CRU, and UDel datasets compared to the mTm at 16 station locations obtained from CRU TS v4.01. The red vertical line in each plot panel represents the optimal value of the corresponding index.

In this step, the monthly mean temperatures (mTm) of CNE datasets were compared with the CPC, CRU and UDel datasets in terms of their capability to reproduce monthly temperatures at 16 stations obtained from the CRU TS v4.01^26^. Data from 10 out of 16 stations were not used during the development of CNE data (*refer to* Fig. 2). As shown in Fig. 7, the RMSE of mTm of CNE was much better than those of CPC, CRU and UDel datasets, with a median of 0.7. The median %BIAS of CNE was 0.7%, while they were 2.15%, −0.7%, and −18.05% for the CPC, CRU, and UDel datasets, respectively. The NSE of CNE was found to be better than that of CPC, CRU, and UDel. In terms of md, the CNE was also found to outperform the others. It also showed a perfect R^2^ (near 1) at all stations, while the median R^2^ of CRU was slightly higher than that of CNE.

### Validation of the spatial variability

**Fig. 8:**
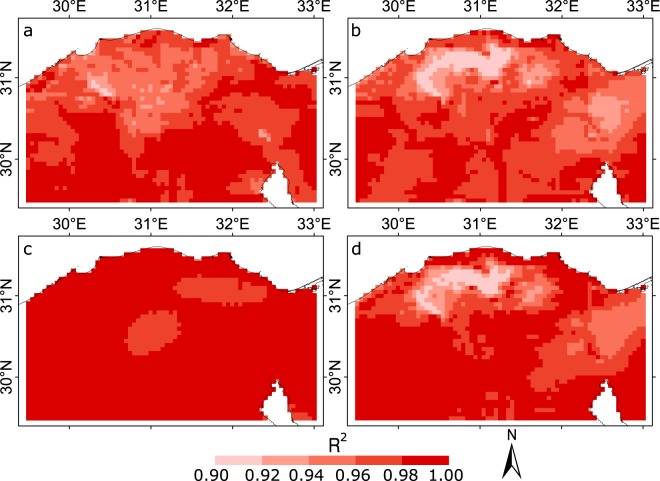
The validation of monthly time series and climatologies of CNE datasets using CHELSA. The spatial patterns in the coefficient of determination (R^2^) between the (**a**) mTmx and (**b**) mTmn time series and the monthly climatologies of (**c**) maximum and (**d**) minimum temperature of the CNE and CHELSA datasets. The results show a high spatial correlation and indicate the ability of CNE to predict the spatial distribution of temperature.

The spatial variability in the CNE datasets was obtained from the WorldClim v.2 after adjusting the WorldClim overall grid monthly means with the station data mean. To validate the spatial variability in the CNE datasets, the monthly time series and climatology of each grid point of the CNE were compared to the corresponding grid point of CHELSA. The CHELSA is an independent dataset that has been developed from ERA-Interim reanalysis data^19^. Figure 8 presents the spatial distribution of R^2^ values estimated for the mTmx and mTmn time series of CNE and CHELSA. The correlation between the monthly climatologies of CNE and CHELSA is also presented in the figure. The R^2^ values were between 0.91 and 1 and between 0.9 and 1 for mTmx and mTmn, respectively. In addition, they were between 0.97 and 1 and between 0.90 and 1 for the Tmx and Tmn monthly climatologies, respectively. The high spatial correlation of the CNE with CHELSA datasets indicates that the CNE datasets are able to predict the spatial distribution of temperatures well.

## Usage Notes

The CNE datasets can be used for many applications at various temporal resolutions. As shown in the validation process, the CNE datasets can estimate hot and cold temperature extremes more accurately than any other datasets in the study region. Furthermore, the high-resolution CNE datasets can be combined with various datasets having the same resolution, including Climate Hazards InfraRed Precipitation with Stations (CHIRPS)^36^, to widen the range of applications of the datasets for greater scientific and social benefits.

## Supplementary Information

### ISA-Tab metadata file

Download metadata file

### Supplementary information

Supplementary Table 1

## Data Availability

The code was written using R software, R.3.4, to produce the data. The code is available online within Figshare^13^.
