# Supplementary material for: Development of high-resolution daily gridded temperature datasets for the central north region of Egypt
Source: Sci Data. 2019 Jul 31;6:138. doi: 10.1038/s41597-019-0144-0 (PMC6668447; doi:10.1038/s41597-019-0144-0)
Supplement: Supplementary file 2 — Supplementary Table 1 [file 41597_2019_144_MOESM2_ESM.docx]

### Supplemental Information

**Data Descriptor Title**

**Development of a high-resolution daily gridded temperature datasets for the central north region of Egypt**

**Authors**

Mohamed Salem Nashwan^1,2^, Shamsuddin Shahid^2^, Eun-Sung Chung^3^

**Affiliations**

1. Construction and Building Engineering, College of Engineering and Technology, Arab Academy for Science, Technology and Maritime Transport (AASTMT), Cairo, 2033 - Elhorria, Egypt.

2. School of Civil Engineering, Faculty of Engineering, Universiti Teknologi Malaysia (UTM), 81310 Johor Bahru, Malaysia.

3. Seoul National University of Science and Technology, Nowon-gu, 01811, Seoul, South Korea.

The content of this file is the Supplementary Table 1.

Supplementary Table 1. The formula used for the estimation of the indices and range of the index values.

| Index formula | Index range |
| --- | --- |
| $RMSE= \sqrt{\frac{1}{n}\sum_{i=1}^{n} \left( T_{m,i}-T_{o,i} \right)^{2}}$ | 0 to +∞ |
| $Pbias= \frac{\bar{T_{m}}- \bar{T_{obs}}}{\bar{T_{obs}}} \times100$ | -∞ to +∞ |
| $NSE=1-\frac{\sum_{i=1}^{n} \left( T_{m,i}-T_{obs,i} \right)^{2}}{{\sum_{i=1}^{n} \left( T_{obs,i}-\bar{T_{obs}} \right)}^{2}}$ | −∞ to 1 |
| $md=1-\frac{\sum_{i=1}^{n} \left( T_{obs,i}-T_{m,i} \right)^{j}}{\sum_{i=1}^{n} \left( \left\vert T_{m,i}- \bar{T_{obs}} \right\vert+\left\vert T_{obs,i}-\bar{T_{obs}} \right\vert\right)^{j}}$ | 0 to 1 |
| $R^{2}=\left( \frac{\sum_{i=1}^{n} \left( T_{obs,i}-\bar{T_{obs}} \right)\left( T_{m,i}-\bar{T_{m}} \right)}{\sqrt{{\sum_{i=1}^{n} \left( T_{obs,i}-\bar{T_{obs}} \right)}^{2}}\sqrt{{\sum_{i=1}^{n} \left( T_{m,i}-\bar{T_{m}} \right)}^{2}}} \right)^{2}$ | 0 to 1 |

*n* is the sample number; $T_{obs,i}$ and $T_{m,i}$refer to the observed and modelled temperature data, respectively; $\bar{T_{obs}}$and $\bar{T_{m}}$ are the mean of the observed and gridded temperature data, respectively.
